# A phase 1, open-label, dose-escalation study of BIIB022 (anti-IGF-1R monoclonal antibody) in subjects with relapsed or refractory solid tumors

**DOI:** 10.1007/s10637-014-0064-y

**Published:** 2014-01-24

**Authors:** Margaret von Mehren, Carolyn D. Britten, Peter Pieslor, Wayne Saville, Artemios Vassos, Sarah Harris, Gerald R. Galluppi, Mohamed Darif, Zev A. Wainberg, Roger B. Cohen, Stephen Leong

**Affiliations:** 1 Fox Chase Cancer Center, Philadelphia, PA USA; 2 University of California at Los Angeles Comprehensive Cancer Center, Los Angeles, CA USA; 3 Present Address: Medical University of South Carolina, Charleston, SC USA; 4 Biogen Idec Inc., Cambridge, MA USA; 5 Present Address: Amgen, Thousand Oaks, CA USA; 6 Present Address: Genoptix, Carlsbad, CA USA; 7 Present Address: Novartis, La Jolla, CA USA; 8 Present Address: Sangrat, San Diego, CA USA; 9 Present Address: Abramson Cancer Center, University of Pennsylvania Medical Center, Philadelphia, PA USA; 10 University of Colorado Cancer Center, Aurora, CO USA

**Keywords:** IGF-1R, Antibody, Phase I, Sarcoma, FDG-PET

## Abstract

*Purpose* The IGF-1R signaling pathway has been implicated in multiple cancers as important for cell survival, proliferation, invasion and metastasis. BIIB022 is a non-glycosylated human IgG4 monoclonal antibody (mAb) with specificity for IGF-1R. Unlike other anti-IGF1R antibodies, BIIB022 has no effector functions. Additionally, inhibition is via an allosteric rather than competitive mechanism, which further differentiates this antibody from others. We sought to determine the safety and tolerability of BIIB022 and determine the pharmacokinetic (PK) and pharmacodynamic (PD) profile of this antibody. *Methods* A multi-institutional phase I study evaluated the safety of escalating doses of BIIB022 given IV q3wk until progression or unacceptable toxicity in patients with advanced solid tumors. Five sequential BIIB022 dose cohorts were evaluated using a standard 3 + 3 dose-escalation design (1.5, 5. 10, 20, 30 mg/kg); 10 additional patients were treated at the recommended phase 2 dose. *Results* 34 patients were treated. Toxicities were manageable and mostly low grade; grade 3–4 hyperglycemia was not observed. No RECIST responses were observed, although three patients had metabolic responses associated with prolonged stable disease. The PK of BIIB022 was nearly linear in the dose range from 10 to 30 mg/kg, with some nonlinearity at lower doses (1.5–5.0 mg/kg), likely due to target-mediated drug disposition of BIIB022 at low serum concentrations. PD analyses showed decrease in IGF-1R levels on leucocytes, with stable serum values of IGF-1 and IGF-2. *Conclusions* BIIB022 can be safely given at 30 mg/kg IV every 3 weeks with preliminary evidence of biological activity in selected patients.

## Introduction

The IGF-1R signaling pathways is critical for cancer survival and proliferation as evidenced by decreased tumor cell growth, survival, motility and invasion when it is blocked [1–3]. Individuals with higher than normal circulating levels of IGF-1 have an elevated risk of developing breast, prostate, lung, and colon cancers [3]. In addition, elevated levels of the IGF-1R ligands, IGF-1 and IGF-2, have been found in patients with breast cancer [4, 5], and in the tumor microenvironment in colorectal tumors [6, 7]. Perturbation with an anti-IGF-1R antibody of IGF-1R axis is therefore hypothesized to have therapeutic value.

BIIB022 is a non-glycosylated human IgG4 monoclonal antibody with specificity for IGF-1R. It inhibits the binding of both IGF-1 and IGF-2 to the receptor via an allosteric mechanism, interacting with an epitope on the fibronectin III-1 domain of IGF-1R that is distinct from the IGF-1 and IGF-2 binding region [8]. BIIB022 blocks both IGF-1 and IGF-2 induced phosphorylation of IGF-1R and downstream substrates, thereby inhibiting ligand-mediated receptor signaling. Additionally, BIIB022 was designed without Fc effector function and C1q binding and therefore cannot activate antibody-dependent cell-mediated cytotoxicity (ADCC) or complement-dependent cytotoxicity (CDC), features that may limit toxicity associated with this agent. *In vitro* studies demonstrated evidence of decreased cell growth following treatment with BIIB022 in lung, pancreas and colon cancer cell lines in the presence of IGF-1 or IGF-2 in culture media [9].

We performed a multi-institutional phase I study of BIIB022 to determine the maximally tolerated dose (MTD), toxicity profile and pharmacokinetic properties of this antibody in patients with advanced malignancies.

## Materials and methods

### Subject selection

Patients with relapsed or refractory solid tumors age 18 or above were screened for eligibility after providing written informed consent. Patients were required to have at least evaluable disease, life expectancy of 3 months or more and an ECOG score of 0–1. Prior therapy was allowed except for prior anti-IGF-1R therapy or prior anti-cancer therapy within 4 weeks of initiation of BIIB022. Other eligibility criteria included adequate hematologic, renal, and hepatic function; no history of diabetes mellitus; and hemoglobin A1c ≤ 6.

### Study design

This was a multi-institutional phase I study to determine the safety, tolerability, and MTD of BIIB022 by intravenous (IV) infusion every 3 weeks. BIIB022 was produced in Chinese Hamster Ovary cells and formulated as a sterile liquid at a concentration of 10 mg/ml. Subjects were enrolled into five sequential BIIB022 dose cohorts (1.5, 5, 10, 20, 30 mg/kg), with no intra-subject dose escalation. Each subject was evaluated for dose-limiting toxicities (DLTs) during the first 28 days. Enrollment into the next higher dose cohort was not permitted unless 0 of 3 or ≤1 of 6 subjects in the previous cohort had DLTs. Subjects who did not receive at least two initial doses of BIIB022 and did not experience a DLT were replaced. DLT was defined as any clinically significant grade ≥3 toxicity regardless of relatedness to BIIB022, including nausea/vomiting and diarrhea if grade ≥3 despite adequate supportive care measures, or treatment delays of ≥14 days due to toxicity. Toxicities were graded according to NCI CTCAE version 3. The study was amended once to collect additional safety assessments: insulin concentration and C-peptide to evaluate hyperglycemia, creatinine kinase to evaluate for muscle damage, prostate-specific antigen (PSA) to evaluate effects on the prostate as well as additional immunogenicity analyses. Extra electro-cardiograms were added in response to a DLT of QTc prolongation in this study and routine audiometry assessments were added in response to an ototoxicity DLT seen in contemporaneous phase I clinical trials of other anti-IGF-1R antibodies. Therapy with BIIB022 was continued until disease progression, unacceptable toxicity, or subject withdrawal.

The recommended Phase 2 dose (RP2D) was determined by evaluating safety and pharmacokinetic (PK) data after all the cohorts completed enrollment and all subjects had been followed for at least 28 days after their first BIIB022 infusion. The RP2D was defined as the MTD, biologically–effective dose (BED), or 30 mg/kg if the MTD or BED were not reached. The BED was defined as the dose at which BIIB022 serum exposure had reached a plateau in 2 successive dose cohorts (indicating receptor saturating exposure), or the BIIB022 dose resulting in human exposure approximately 10 times higher than serum levels associated with maximal anti-tumor activity in animal xenograft models. An additional 10 subjects were treated at RP2D to further evaluate safety, with emphasis on evaluating any evidence of cumulative toxicity. Secondary endpoints included PK and immunogenicity assessments, as well as efficacy using RECIST. Serum samples for BIIB022 concentration determination and anti-BIIB022 antibody formation were collected after the last dose and once a month for 2 months, as long as clinically indicated. Exploratory objectives included biomarker evaluation of archival tumor tissue and peripheral blood as well as FDG-PET scans.

The study was approved by the institutional review board or independent ethics committee at each of the participating centers, and was performed in accordance with federal and institutional guidelines, observing the standards set by the Helsinki Declaration.

### Anti-tumor activity

Response was determined by imaging studies performed every 2 cycles using RECIST version 1.0. Metabolic response was assessed by FDG-PET at baseline and again between weeks 1 and 3 of treatment utilizing the criteria developed by the European Organization for Research and Treatment of Cancer (EORTC) [10].

### Pharmacokinetics

Blood samples for determining PK were drawn cycle 1 at baseline; 15 and 45 min, 1.5, 3, 5, 8, 24, and 96 h and on days 8 and 15 following completion of the BIIB022 infusion. Sparse sampling for PK was performed at 15 min and days 8 and 15 following all subsequent BIIB022 doses. BIIB022 serum concentrations were determined by an enzyme-linked immunosorbent assay (ELISA), performed at Biogen Idec, (La Jolla, CA). In brief, plates were coated with mouse anti-human IGF-1R followed by a secondary coating of recombinant human IGF-1R. BIIB022 levels were detected with mouse anti-human IgG4-HRP. LLOQ of the assay was 160 ng/ml. Noncompartmental PK analysis was conducted on the concentration-time data for each subject using WinNonlin (Pharsight Co. version 5.2). Statistics were then calculated for the resulting PK parameters in each dose cohort.

### Pharmacodynamics

IGF1R down-regulation was measured on blood granulocytes on Day 1 (pre- and post-infusion), Day 2, and Day 5 of Cycles 1 and 2. Flow cytometric analyses to determine the expression of IGF-1R on the surface of granulocytes were performed at Biogen Idec (La Jolla, CA). The chimeric IgG4.P.agly anti-IGF1R MAb P1E2 was used to assess receptor downregulation: the MAb probe binds to IGF1R on blood granulocytes, and does not compete with BIIB022 allowing qantitation of IGF1R levels in the presence of BIIB022. IGF1 and IGFBP3 levels were measured in the serum using validated in vitro diagnostic approved immunoassay kits from Diagnostic Systems Laboratories Corp. (Webster, TX).

### Statistics

The study was a multi-institutional phase I trial that utilized a standard 3 + 3 dose-escalation design based on the observed toxicities in cycle 1. The study population for safety analyses included all patients who received at least one dose of BIIB022. The DLT-evaluable population included all patients who met DLT assessment criteria as described above. The PK-evaluable population included all patients for whom PK sampling was completed on at least 1 day. The efficacy-evaluable population included all patients with measurable disease at baseline. Descriptive statistics were used to summarize patient characteristics, treatment administration/compliance, safety, PK parameters, and efficacy.

## Results

Thirty-five patients were enrolled at 3 centers in the United States. Thirty-four patients received at least one infusion of BIIB022. Table 1 summarizes the demographics of the treated patients; this was a population with advanced malignancies, 76 % of whom had 2 or more sites of metastases, and 100 % of whom had received prior chemotherapy.

**Table 1:** Patient characteristics

Patient characteristics *N* = 34 (%)	
Age:	
Range: 27–76 year	
Median: 58.5 year	
M/F:	20 (59)/ 14 (41)
ECOG PS	
0:	14 (41)
1:	20 (59)
Cancer type	
Colon:	8 (23)
Lung:	2 (6)
Pancreas:	2 (6)
Prostate:	1 (3)
Sarcoma^a^:	16 (47)
Other^b^:	5 (15)
Stage at study entry	
II:	1 (3)
III:	1 (3)
IV:	31 (91)
Unknown:	1 (3)
Time from diagnosis^c^	
Range: 0.6–13.9 years	
Median: 4.34 years	
Prior chemotherapy	34 (100)
Range: 1–27	
Median: 7.5	

^a^Liposarcoma, *n* = 4; Leiomyosarcoma, *n* = 3; High grade sarcoma NOS, *n* = 2; and one each of the following: adenosarcoma of the uterus, chondrosarcoma, desmoplastic small round cell tumor, Ewing’s Sarcoma/PNET, pleomorphic sarcoma, rhabdomyosarcoma, and spindle cell sarcoma

^b^Other tumor types include adrenal cell carcinoma, ovarian cancer, pulmonary carcinoid tumor, squamous cell carcinoma of tongue, thymoma

^c^Years since diagnosis = (Date informed consent first signed—date of initial diagnosis + 1) / 365.25

### Toxicity

All treated subjects experienced at least 1 adverse event, primarily grade 1 or 2 (see Tables 2 and 3), with 91 % of them reported as related to BIIB022. Related AEs reported in 10 % or more of patients included: headache, fatigue, dizziness, nausea, and muscle spasms. There were no patients with grade 3 or 4 hyperglycemia; in addition there was no evidence for increasing serum insulin or fructosamine levels over time or at increased BIIB022 doses. Twenty-one percent of subjects experienced a Grade 3 treatment-related AE. This included 1 subject in the 1.5 mg/kg dose cohort (deep vein thrombosis); 2 subjects in the 10 mg/kg dose cohort (hypertension and dyspnea); 2 subjects in the 20 mg/kg dose cohort (asthenia and ECG QTc prolonged); and 2 subjects in the 30 mg/kg dose cohort (pulmonary congestion and fatigue in 1 subject and gastrointestinal (GI) hemorrhage in 1 subject). There were no grade 5 DLTs. The four deaths reported on study were due to disease progression.

**Table 2:** Incidence of most common (≥10 %) and all severe AEs related to BIIB022

	Toxicity grade					Total
	1	2	3	4	5	
Headache	18 (53 %)	2 (6 %)	0	0	0	20 (59 %)
Fatigue	4 (12 %)	5 (15 %)	1 (3 %)	0	0	10 (29 %)
Dizziness	2 (6 %)	3 (9 %)	0	0	0	5 (15 %)
Nausea	4 (12 %)	1 (3 %)	0	0	0	5 (15 %)
Muscle spasms	2 (6 %)	3 (9 %)	0	0	0	5 (15 %)
Hypertension	0	1 (3 %)	1 (3 %)	0	0	2 (6 %)
Dyspnea	0	1 (3 %)	1 (3 %)	0	0	2 (6 %)
asthenia	1 (3 %)	0	1 (3 %)	0	0	2 (6 %)
Gastrointestinal hemorrhage	0	0	1 (3 %)	0	0	1 (3 %)
Deep vein thrombosis	0	0	1 (3 %)	0	0	1 (3 %)
Prolonged QTc interval	0	0	1 (3 %)	0	0	1 (3 %)

**Table 3:** All grade 3 AEs related to BIIB022

Adverse event	Relationship to BIIB022	BIIB022 Dose (mg/kg)
DVT	Unlikely	1.5
Hypertension	Possibly	10
Dyspnea	Possibly	10
Asthenia	Unlikely	20
QT prolongation	Possibly	20
Gastrointestinal haemorrhage	Unlikely	30
Fatigue	Possibly	30

There were two patients who experienced DLT. A 57 year old woman with colorectal cancer and no prior history of heart disease treated at 20 mg/kg was found to have an asymptomatic grade 2 T-wave inversion and grade 3 prolonged QTc on ECG on day 26. There was no evidence of ischemia or infarction and an echocardiogram revealed transient decreased apical wall motion. This was judged possibly related to BIIB022, and the patient was removed from study and recovered without sequelae. A total of six patients were treated with BIIB022 20 mg/kg without further DLTs, including ECG changes. In addition, subsequent patients were monitored for QTc prolongation with the median maximum change found to be 25.7 msec from baseline.

The second DLT occurred at the 30 mg/kg dose level in a 26 year old patient with small round blue cell sarcoma. Prior to study entry the patient had experienced bleeding from tumor in the jejunum. On day 22 following his first infusion, the patient developed a grade 3 GI bleed, which was felt to be unlikely related to BIIB022. Although the GI bleed was most likely related to the tumor, a contribution from BIIB022 could not be ruled out, and it was considered a DLT based on the definitions set forth in the protocol. The patient was withdrawn from the study and no further DLTs were noted at this dose level. An additional patient at the 30 mg/kg level was removed from study after being diagnosed with myelodysplastic syndrome. This was not considered secondary to BIIB022 as the patient had received extensive prior therapy with cytotoxic chemotherapies, including very prolonged treatment with doxorubicin.

### Pharmacokinetics

**Fig. 1:**
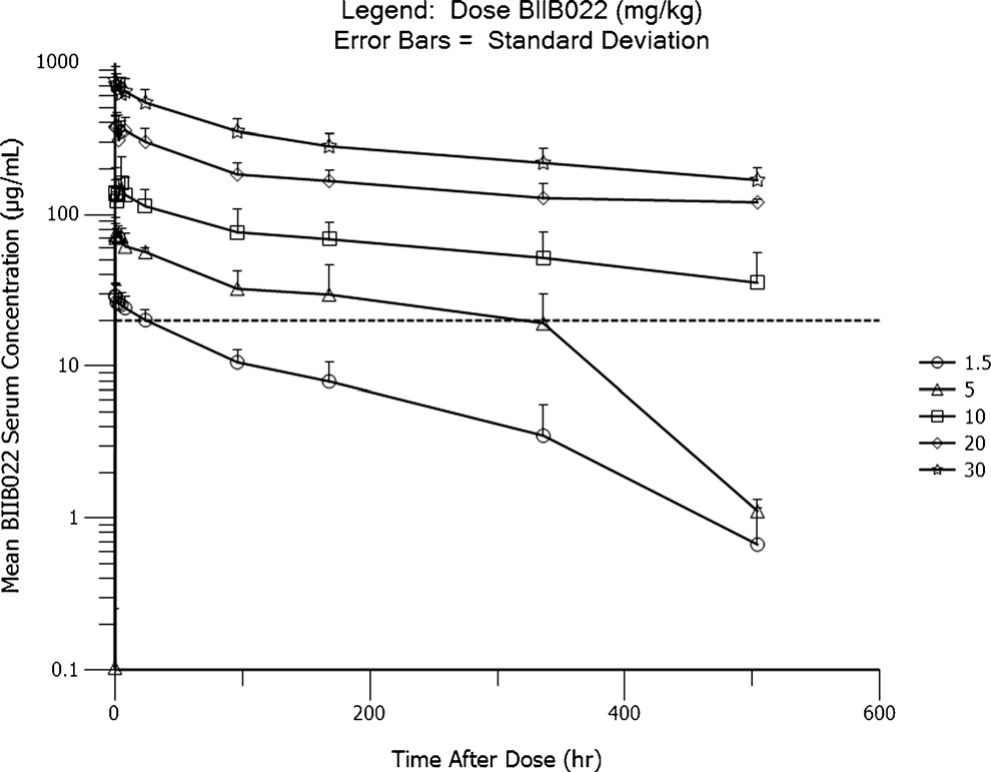
Mean BIIB022 concentration versus time

**Fig. 2:**
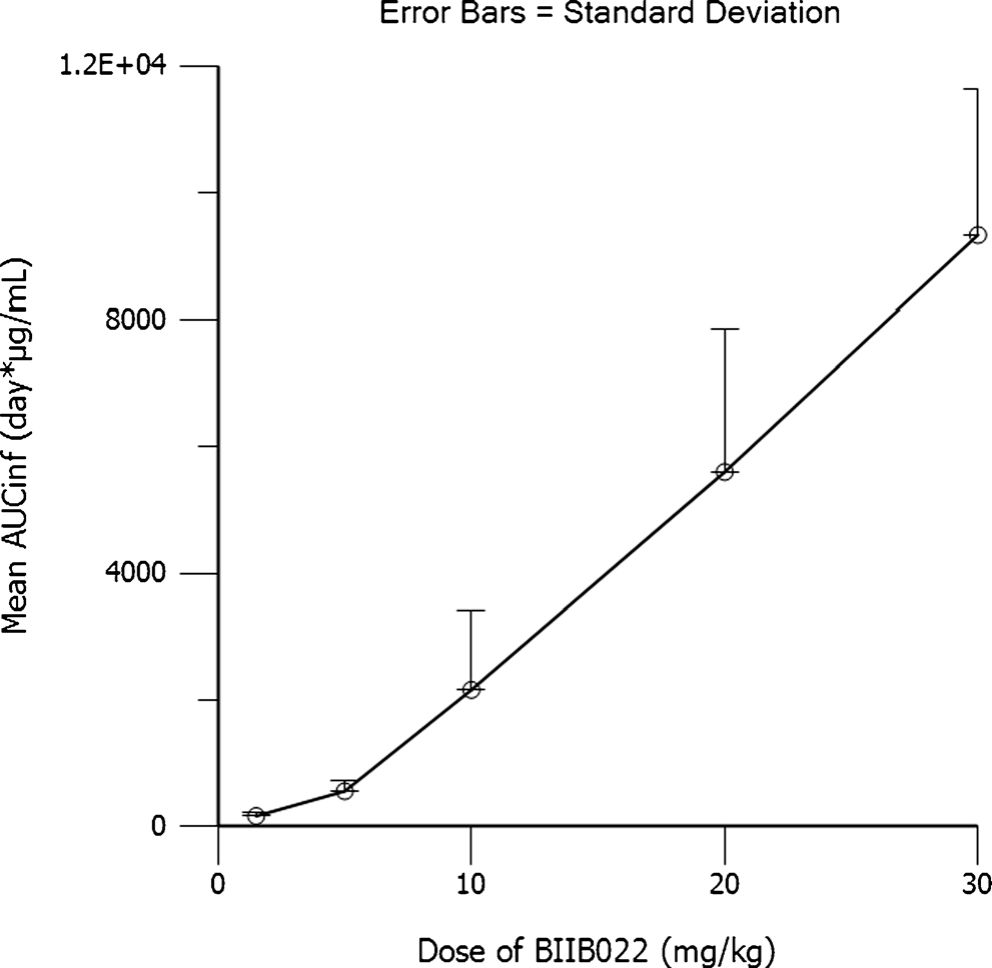
Mean AUC versus dose of BIIB022

Mean BIIB022 serum concentration versus time data for all dose cohorts are plotted in Fig. 1. Serum exposure increased in a dose-dependent manner, but BIIB022 demonstrated nonlinear PK, as indicated by the plot of AUC_inf_ versus Dose in Fig. 2. The steady-state volume of distribution approximated blood volume at all dose levels, which is consistent with distribution volumes reported for other monoclonal antibodies.

### Pharmacodynamic studies

IGF-1R levels declined shortly after BIIB022 dosing in all dose cohorts. There was no apparent dose–response relationship found. IGF-1R levels remained low throughout treatment while granulocyte counts remained relatively constant. In addition, median IGF-1 serum levels showed no change through day 15 of cycle 3 except for in the 10 mg/kg dose cohort, which likely reflected the effect of one outlier. Mean levels of IGF-BP3 serum levels were also unchanged through day 15 of cycle 3.

### Anti-tumor activity

Patients were evaluated for efficacy by investigators every 5–6 weeks using RECIST (version 1.0). Efficacy data are available on 24 pts (3 in 1.5 mg/kg cohort, 2 in 5 mg/kg cohort, 3 in 10 mg/kg cohort, 5 in 20 mg/kg cohort, and 11 in 30 mg/kg cohort). There were no complete or partial responses. Fifty nine percent of patients (*n* = 20) had stable disease as best response, while 29 % (*n* = 10) had progressive disease and 12 % (*n* = 4) were not evaluable for response. Six patients with sarcoma remained progression free for 3.75 (30 mg/kg), 4.5 (2 patients at 30 mg/kg), 5.25 (5 mg/kg), and 6 months 1.5 mg/kg and 20 mg/kg; of these, 3 patients had retroperitoneal liposarcoma.

Metabolic response was also assessed by FDG-PET scan at baseline and during treatment (1 week after 1st infusion and before 3rd infusion). Thirty-two patients had PET imaging at baseline and during cycle 1 (day 8) and prior to cycle 3. Twenty-eight percent (9 of 32) had a decline in SUV of >25 % (see Table 4). The median number of cycles on study in patients with PET responses was 7 versus those patients with stable or progressive disease in which the median number of cycles was 2.5.

**Table 4:** PET response data

Dose level mg/kg	Diagnosis	Baseline max SUV	Cycle 1 Max SUV (% change from baseline)	Cycle 3 Max SUV (% change from baseline)	Number of cycles on study
1.5	Retroperitoneal sarcoma	15.90	10.50 (−33.96)	9.9 (−37.74)	8
5.0	Synovial sarcoma	1.85	1.25 (−34.83)	1.40 (−26.67)	7
20.0	Rhabdomyosarcoma	6.15	4.80 (−22.12)	4.25 (−31.71)	4
20.0	Adrenal cortical Ca	12.7	9 (−29.13)	12.3 (−0.03)	10
30.0	Spindle cell sarcoma	7.50	4.70 (−38.74)	4.25 (−44.71)	5
30.0	Ewing’s sarcoma	6.55	3.30 (−50.47)	4.13 (−37.96)	3
30.0	Adenosarcoma of uterus	5.30	3.05 (−42.69)	4.70 (−11.13)	4
30.0	High grade spindle cell sarcoma NOS	3.55	ND	2.15 (−39.74)	7

Notably, 7 of these 9 patients with SUV declines of >25 % were diagnosed with sarcoma. Metabolic responses correlated with disease stabilization for more than 4 months in 4 of these patients. The first was a 58-year old male with Stage IV liposarcoma enrolled in 1.5 mg/kg cohort. FDG uptake on cycle 1, day 8 had decreased by 34 % from baseline. He received 10 infusions of BIIB022 and maintained stable disease for 7 months. A second patient was a 66 year old male also with metastatic retroperitoneal liposarcoma. FDG uptake on cycle 1, day 8 demonstrated a mixed response with one stable lesion and another with a 38 % increase in SUV. A repeat study cycle 2 day 20 demonstrated a 31 % decrease in the initially stable lesion and a return to baseline of the lesion of the second lesion. He received 7 months of 20 mg/kg BIIB022 before progressing by RECIST. The third patient was a 59-year old male with spindle cell carcinoma who was enrolled in the 30 mg/kg cohort. His FDG uptake on day 43, after 2 BIIB022 doses, had decreased by 40 % compared with baseline. He received 7 infusions of BIIB022 and had stable disease for 4 months. Lastly, a patient with adrenal carcinoma at the 30 mg/kg cohort remained on study for 10 cycles. His FDG uptake on cycle 1 day 8 had decreased by 30 % compared to baseline.

## Discussion

In this phase I trial of BIIB022 monotherapy, we found that therapy was generally well-tolerated at doses up to 30 mg/kg given every 3 weeks in patients with relapsed and refractory solid tumors. A low incidence of grade 3 toxicities was observed, and no grade 4 or 5 toxicities. There was preliminary evidence of biologic activity; more than in 25 % of patients had metabolic responses and 8 patients were without progressive disease for 3.75 months (5 cycles) or longer.

BIIB022 has a unique structure compared with other antibodies targeting IGF-1R. Ganitumab (AMG-479), R1507, and daltuzumab (MK-0646) are humanized IgG1 antibodies while figitumimab (CP-751,871) is a humanized IgG2 antibody [11–13]. By contrast, BIIB022 is a non-glycosylated human IgG4 monoclonal antibody that lacks Fc effector and C1q binding domains. Potential benefits of this unique structure are improved toxicity because of lack of CDC, which was supported by the absence of any observed infusion reactions in our trial. All the IGF-1R targeting antibodies have been shown to bind to IGF-1R. Daltuzumab is the only agent reported to be able to bind to IGF-1R hybrid receptors. Once these antibodies bind IGF-1R or hybrid receptors, they prevent binding of the ligands IGF-1 and IGF-2 (R1507 does not block IGF-2) thereby blocking ligand induced phosphorylation of the receptor. Unlike the other antibodies in development, BIIB022 interferes with receptor function via an allosteric mechanism, as it binds an epitope on the fibronectin III-1 domain of IGF-1R that is distinct from the IGF-1 and IGF-2 binding regions [8]. Thus, BIIB022 can engage IGF-1R in the presence of ligands and is hypothesized to cause a conformational change that significantly reduces ligand affinity for the receptor.

Toxicities observed in our study were consistent with those previously reported with this class of therapeutic agents. While toxicities (all grades) were reported in all patients, the proportion of patients with grade 3 toxicities related to BIIB022 was only 21 %. Unlike reports from other phase I studies of IGF-1R targeting antibodies, we did not observe any hyperglycemia, although we excluded patients with a history of diabetes mellitus or patients with baseline hemoglobin A1c of >6 %, a lower cut-off than other studies that used 8 % as the eligibility criterion. We did note some cardiovascular effects that may have been attributable to BIIB022, including one patient with hypertension and a second with asymptomatic EKG changes including T-wave inversion and QTc prolongation. IGF-1 has been shown in preclinical models to aid in survival of myocardium in the setting of ischemia. Consequently, our clinical findings cannot exclude a causal relationship to BIIB022 therapy. A deep venous thrombosis was noted in one of our patients, which is not uncommon in patients with advanced malignancies. We note that cerebral ischemia was reported in a patient treated with R1507. It seems unlikely that either of these events were drug related as there are no data to date showing that inhibition of IGF-1R perturbs coagulation and patients with metastatic cancers are known to be hypercoagulable.

The pharmacokinetic data demonstrated a dose dependency, but were not linear. The nonlinearity in PK at the lower doses is likely due to target-mediated disposition of BIIB022 at serum concentrations below 20 μg/mL denoted by the horizontal line on Fig. 1. At higher concentrations, we hypothesize that target-mediated pathway becomes saturated, and the non-specific FcRN-mediated clearance pathway predominates. Consistent with this hypothesis, the data in Table 5 shows that the apparent clearance of BIIB022 was 0.69–0.73 L/day at the lower dose levels but settled into a range from 0.26 to 0.33 L/day at doses of 10 to 30 mg/kg. Likewise, the mean observed terminal t_1/2_ increased from 4.7 to 5.1 days at the lower doses to 13.7–15.4 days at 10 to 30 mg/kg, consistent with a shift from target-mediated clearance at the lower dose levels to nonspecific clearance at the higher dose levels. In the linear PK range the clearance of BIIB022 was 0.27–0.33 L/day, the steady-state distribution volume was 4.9–6.0 L, and the terminal elimination half-life was 14–15 days. The PK characteristics of BIIB022 are typical of those reported for other monoclonal antibodies. The terminal half-life supports a 3-week dosing schedule for chronic administration of BIIB022.

**Table 5:** Pharmacokinetic parameters

Pharmacokinetic parameter^a^	BIIB022 dose (mg/kg)				
	1.5 (*N* = 4)	5 (*N* = 3)	10 (*N* = 3)	20 (*N* = 6)	30 (*N* = 18)
C_max_ (μg/mL)	29.8 ± 6.13	82.6 ± 7.13	172 ± 70.7	412 ± 78.2	801 ± 48.0
AUC_inf_ (day^a^μg/mL)	164 ± 45.3	559 ± 166	2160 ± 1260	5610 ± 2260	9330 ± 2310
CL (L/day)	0.686 ± 0.107	0.734 ± 0.267	0.327 ± 0.078	0.268 ± 0.108	0.262 ± 0.085
t_1/2_ (day)	4.72 ± 0.401	5.12 ± 2.01	13.7 ± 5.24	15.2 ± 6.64	15.4 ± 5.71
V_ss_ (L)	4.18 ± 0.519	5.37 ± 1.03	6.00 ± 1.36	4.94 ± 0.221	5.34 ± 2.23

^a^Data presented are the mean ± standard deviation from dose 1 only

*C*_*max*_ maximum-observed serum concentration, *AUC* area-under-the concentration time curve, *CL* clearance, *t*_*1/2*_ terminal half-life, *V*_*ss*_ volume of distribution at steady-state

We did not observe any RECIST responses, although 9/32 (28 %) patients treated with BIIB022 had a metabolic response as defined by the EORTC criteria. Interestingly, 4 of these patients also had prolonged stable disease, 3 of whom were treated at 20 mg/kg. Although a quantitative link between BIIB022 exposure required for efficacy in animals and humans has not been established, at a dose of 20 mg/kg IV q 3 weeks, BIIB022 achieved trough serum levels in human subjects comparable to levels that consistently demonstrated anti-tumor activity in several mouse xenograft tumor models. Other IGF-1R inhibitor studies have demonstrated mixed or partial responses in patients with Ewing’s Sarcoma and neuroendocrine tumors [11–15]. In our patient population we did not have any patients with neuroendocrine tumors. Our largest group of patients on study had a diagnosis of sarcoma (Table 1). We only treated 1 patient with Ewing’s Sarcoma/PNET, who received 4 cycles prior to disease progression. A second patient with a small round blue cell tumor experienced the tumor-related gastrointestinal bleed and DLT. Of note we did see prolonged stable disease in 3 of 6 liposarcoma patients which is interesting as IGF-1R has been reported as a potential therapeutic target in liposarcomas [16].

Biomarker studies demonstrated down regulation of IGF-1R levels on blood granulocytes, with no significant changes in the levels of IGF-1R’s stimulatory and inhibitory ligands IGF-1 and IGFBP-3. This is in contrast to other antibodies where an increase in IGF-1 has been documented [11–13]. For example, at the 9 mg/kg dose of R1507, IGF-1 increased by 250 % from baseline and after 7 doses the mean percentage ranged from 100 to 350 % for all dose levels[12]. The only study reporting levels of the inhibitory ligand IGF-BP3 also noted an increase in these levels[13]. The difference in the levels of stimulatory or inhibitory ligands is of unclear significance. Studies of other targeted therapies have reported increases in the concentration of receptor ligands over time [17, 18]. A key difference may be that BIIB022, unlike all the other IGF-1R antibodies, is not a competitive inhibitor of the ligand binding sites, which might explain the lack of alteration of these levels. An alternative explanation of the lack of effect on ligands is that BIIB022 for some reason was not having a therapeutic effect. Against this interpretation, however, is the observed decrease in IGF-1R levels on PMNs as well as the metabolic responses on PET scans. We only collected serum levels of ligands and therefore cannot comment on effects on intratumoral ligand levels, which would require on-study biopsies.

In conclusion, BIIB022 was found to be well tolerated at doses ranging from 1.5 to 30 mg/kg given intravenously every 3 weeks. The toxicity profile was similar to other anti-IGF-1R mAbs with the exception of hyperglycemia, which was not observed in our study, and our observation of prolonged QTc associated with a transient wall motion abnormality in one patient receiving BIIB022. In general, the therapeutic benefits of IGF-1R inhibitors have been disappointing, but there have been some limited responses and instances of disease stabilization in patients with sarcomas [12, 14, 15, 19]. This suggests that IGF-1R inhibitors may yet play a role in the anti-cancer armamentarium, if a reliable biomarker of disease sensitivity can be found in order to allow for rational patient selection and optimization of the risk:benefit ratio.
